# An Update on the Role of the Actin Cytoskeleton in Plasmodesmata: A Focus on Formins

**DOI:** 10.3389/fpls.2021.647123

**Published:** 2021-02-15

**Authors:** Min Diao, Shanjin Huang

**Affiliations:** ^1^Center for Plant Biology, School of Life Sciences, Tsinghua University, Beijing, China; ^2^iHuman Institute, Shanghai Tech University, Shanghai, China

**Keywords:** intercellular trafficking, plasmodesmata, actin, actin-binding protein, formin

## Abstract

Cell-to-cell communication in plants is mediated by plasmodesmata (PD) whose permeability is tightly regulated during plant growth and development. The actin cytoskeleton has been implicated in regulating the permeability of PD, but the underlying mechanism remains largely unknown. Recent characterization of PD-localized formin proteins has shed light on the role and mechanism of action of actin in regulating PD-mediated intercellular trafficking. In this mini-review article, we will describe the progress in this area.

## Introduction

The growth and development of multicellular organisms requires intercellular communication. Intercellular communication in plants can be classified into symplasmic and apoplasmic pathways. For the symplasmic pathway, intercellular communication is achieved through complex channel-like structures embedded within the cell walls, called plasmodesmata (PD). The development of the PD structure enables the trafficking of molecules between adjacent plant cells, including some small molecules, such as ions, carbohydrates, and hormones, as well as some large molecules including RNAs, proteins, and viruses (Tilsner et al., 2016; Lee and Frank, 2018). As such, PD are involved in the regulation of plant growth and development and environmental adaptation including disease resistance (Cheval and Faulkner, 2018). The structure and function of PD must be tightly regulated throughout the life of a plant (Lee and Frank, 2018). Indeed, many factors have been shown to be involved in regulating the permeability of PD. For instance, the callose at the neck of PD is involved in the regulation of intercellular trafficking in plants. It was shown that callose deposition at PD will accelerate during virus infection in order to prevent the spread of viruses (Levy et al., 2007). In line with this finding, some viruses have movement proteins (MPs), which can mediate the degradation of callose to open up PD (Schoelz et al., 2011). In addition, consistent with the presence of actin cytoskeletal proteins in PD, the actin cytoskeleton has been implicated in the regulation of intercellular trafficking *via* PD (White and Barton, 2011; Pitzalis and Heinlein, 2017), but the underlying mechanism remains largely unexplored. In this mini-review, we are going to describe the recent progress made in this respect.

## Evidence Supporting the Role of Actin in Regulating the Permeability of PD

Actin is a highly conserved 42 kDa protein, and it is very abundant in eukaryotes. Actin is involved in many cellular physiological processes in plants, including cell growth, cell division, cytokinesis, and various intracellular trafficking events. As such, actin plays a crucial role in plant growth and development (Szymanski and Staiger, 2018). Under optimal conditions, actin can assemble into filamentous structures, called actin filaments (F-actin) or microfilaments. Most actin-based functions are dictated by the spatial organization and dynamics of F-actin in cells. Within cells, actin is associated with many proteins, called actin-binding proteins (ABPs), which modulate the kinetics of actin assembly and disassembly as well as facilitating the formation of different actin structures (Wang et al., 2015). Characterization of the role and mechanism of action of ABPs promises to provide insights into the action of actin within different cellular physiological processes.

Experimental treatments with actin-based pharmacological agents showed that the actin cytoskeleton is involved in the regulation of intercellular communication *via* PD. It was shown that the transport efficiency through PD increases after microinjection of specific actin depolymerizers into tobacco mesophyll cells, whereas the transport efficiency decreases after microinjection of the microfilament stabilizer phalloidin into the cells (Ding et al., 1996; Su et al., 2010). In line with these findings, treatment with the myosin inhibitor 2,3-butanedione monoxime (BDM) reduces the neck width of PD (Radford and White, 1998). However, given that those drugs non-selectively target the actin cytoskeletal system within cells, it remains uncertain whether, and to what extent, the changes in structure and function of PD result from the alteration in the actin cytoskeletal system.

In addition to functioning in plant growth and development, PD are involved in defense against plant pathogens (Cheval and Faulkner, 2018). The important role of PD in virus infection is quite obvious, as viruses spread between cells using PD as the channels. Plant viruses encode MPs to mediate the intercellular transport of infectious genomes *via* PD. It was reported that MPs can mediate the degradation of callose to open up PD (Schoelz et al., 2011). Besides that, another interesting report showed that MPs open up PD *via* interacting with the actin cytoskeleton in PD. Specifically, it was shown that *Cucumber mosaic virus* (CMV) MP severs and caps actin filaments *in vitro* and its filament severing activity is required for its function in PD (Su et al., 2010). Accordingly, it was shown that pretreatment with the actin monomer sequestering reagent latrunculin A (LatA) to depolymerize actin filaments promotes the function of MP in opening up PD, whereas pretreatment with phalloidin to stabilize actin filaments has the opposite effect (Su et al., 2010). These studies imply that there might exist endogenous ABPs that are involved in regulating the permeability of PD *via* controlling actin dynamics in PD. However, due to the lack of techniques to directly visualize the actin cytoskeleton in PD, there is still a debate about whether filamentous actin exists in PD and, if so, how it is organized. This prevents us from understanding the function of the actin cytoskeleton in regulating cell-to-cell trafficking *via* PD. In this regard, development of technology enabling the visualization of the actin cytoskeleton in PD is extremely necessary. In addition, development of methods to specifically alter actin dynamics in PD might provide insights into the function and mechanism of action of actin in the regulation of PD function.

## The Presence of Actin and Actin-Binding Proteins in PD

The involvement of the actin cytoskeleton in regulating the function of PD is also supported by data showing that actin and some ABPs associate with PD. The association of actin with PD was initially discovered by the immunogold labeling approach (Table 1; White et al., 1994; Blackman and Overall, 1998) using a monoclonal antibody against chicken gizzard actin. The association of actin with PD structures was further confirmed using fluorescent phalloidins or by immunofluorescence using an antibody against human actin (Table 1; Baluska et al., 2001, 2004).

**Table 1:** Actin and its associated proteins identified in plasmodesmata (PD).

Cytoskeletal protein	Function	Reference(s)
Actin	Building blocks of the actin cytoskeleton	White et al., 1994; Ding et al., 1996; Blackman and Overall, 1998; Fernandez-Calvino et al., 2011
Myosin	Actin filament side binding; actin-based movement	Blackman and Overall, 1998; Radford and White, 1998; Reichelt et al., 1999; Volkmann et al., 2003; Wojtaszek et al., 2005; Golomb et al., 2008; Sattarzadeh et al., 2008; Fernandez-Calvino et al., 2011; Haraguchi et al., 2014
Tropomyosin	Actin filament side binding	Faulkner et al., 2009; Fernandez-Calvino et al., 2011
ARP2/3	Actin nucleation	Van Gestel et al., 2003
NET	Actin binding	Deeks et al., 2012
Formin	Barbed end capping, actin nucleation	Diao et al., 2018; Oulehlova et al., 2019
Profilin	Actin monomer binding	Fernandez-Calvino et al., 2011
ADF	Actin filament severing; actin monomer binding	Fernandez-Calvino et al., 2011
GSD1	Actin binding	Gui et al., 2014

Similarly, myosin was first discovered to associate with PD with immuno-EM using polyclonal antibodies against animal myosins (Table 1; Blackman and Overall, 1998; Radford and White, 1998), which recognize highly conserved motifs in the myosin head, as well as an antibody against the C-terminal tail of plant myosin VIII (Table 1; Reichelt et al., 1999). The association of myosins with PD was also verified by immunofluorescence analyses with the same antibodies (Table 1; Radford and White, 1998; Reichelt et al., 1999; Baluska et al., 2001, 2004). Subsequent analysis of myosin XI fused to different fluorescent proteins showed no localization to PD (Reisen and Hanson, 2007). Interestingly, one GFP fusion with the IQ-tail zone of ATM1, a member of the *Arabidopsis* myosin VIII family, appears to localize to sites of ER attachment as well as pitfields when expressed in *Nicotiana benthamiana* leaves (Golomb et al., 2008).

In addition, it was shown that tropomyosin-like proteins localize to PD and cell plates using antibodies against mammalian tropomyosins (Table 1; Faulkner et al., 2009). Using the same approach, it was shown that actin-related protein 3 (Arp3) is localized in PD and multivesicular bodies (MVBs) in maize and tobacco (Table 1; Van Gestel et al., 2003). In addition, it was shown that a plant-specific ABP, network protein 1A (NET1A), is able to localize to PD (Table 1; Deeks et al., 2012). Another interesting report showed that grain setting defect1 (GSD1), a plant-specific remorin protein, is able to interact with actin (Gui et al., 2015) and can localize to PD (Table 1; Gui et al., 2014). The presence of ABPs in PD was also supported by data showing that profilin and ADF are present in the *Arabidopsis* plasmodesmal proteome (Table 1; Fernandez-Calvino et al., 2011). Certainly, direct cytological evidence is needed to confirm that these proteins are indeed localized to PD. Interestingly, recent characterization showed that several *Arabidopsis* and rice class I formins associate with PD (Table 1; Diao et al., 2018; Oulehlova et al., 2019). In summary, actin and some ABPs are able to associate with PD.

## The Role of Class I Formins in Regulating the Permeability of PD

Formin (formin homology protein) nucleates actin assembly for the generation of linear actin bundles. The formin proteins contain the characteristic formin homology domain 1 (FH1) and FH2, which are capable of nucleating actin assembly from actin or actin-profilin complexes. The biochemical activities of plant formins have been characterized extensively *in vitro* and most of them are typical formins that nucleate actin assembly from actin or actin bound to profilin (van Gisbergen and Bezanilla, 2013). *In vitro* biochemical analysis revealed that some plant formins have evolved some unusual activities. For instance, AtFH1 was shown to be a nonprocessive actin polymerase, which can bundle actin filaments (Michelot et al., 2006). The formin proteins have been implicated in numerous actin-based cellular processes in plants, such as pollen germination (Lan et al., 2018; Liu et al., 2018), polarized pollen tube growth and root hair growth (Ye et al., 2009; Cheung et al., 2010; Huang et al., 2013; Lan et al., 2018), cell division (Li et al., 2010), cytokinesis (Ingouff et al., 2005), and cell expansion (Yang et al., 2011; Zhang et al., 2011), as well as defense (Favery et al., 2004). There are 21 formin genes in the *Arabidopsis* genome, and the encoded proteins can be divided into two classes (Blanchoin and Staiger, 2010). Specifically, there are 11 class I formins and 10 class II formins in *Arabidopsis*. Among them, Class I formins contain the characteristic transmembrane domain (TMD) at their N-terminus, which enable them to target to membranes (van Gisbergen and Bezanilla, 2013).

Interestingly, recent studies showed that several class I formins specifically localize to PD (Diao et al., 2018; Oulehlova et al., 2019) and they are involved in regulating the permeability of PD in *Arabidopsis* (Diao et al., 2018). It was shown that the class I formin AtFH2 localizes to PD in various tissues, and this function is dictated by its N-terminal TMD. Analysis of *atfh2* mutants showed that the permeability of PD is increased when compared to WT. As such, *atfh2* mutants are sensitive to virus infection. Strikingly, it was shown that a mutant AtFH2, which was deficient in interacting with actin filaments, failed to rescue the defective intercellular trafficking *via* PD in *atfh2* mutants. This suggests that the interaction of AtFH2 with the actin cytoskeleton is crucial for its function in PD. *In vitro* biochemical analysis showed that AtFH2 lacks actin nucleation activity but it caps the barbed end of actin filaments and stabilizes them against dilution-mediated depolymerization *in vitro* (Diao et al., 2018). This allows us to speculate that actin filaments become instable and/or the amount of actin filaments is reduced in PD in *atfh2* mutants. It is quite unusual that AtFH2 can cap the barbed end of actin filaments to prevent their elongation but fails to nucleate actin assembly *in vitro*. Certainly, it cannot be completely ruled out that AtFH2 is able to nucleate actin assembly after post-translational modification or by interacting with some partners *in vivo*. Nonetheless, the current *in vitro* biochemical data suggest that AtFH2 regulates actin dynamics only by binding to the barbed end of filamentous actin. To some extent, this supports the notion that actin filaments exist in PD. Certainly, uncovering the precise localization of AtFH2 in PD will further refine this hypothesis. However, we still do not know how to fit actin filaments into PD as the gap between the plasma membrane and the ER (called the cytoplasmic sleeve) within PD pores is less than 10 nm (Nicolas et al., 2017). It could be possible that actin filaments stay in cytoplasmic sleeve but twine around the ER within PD pores. In addition, Nicolas et al. (2017) also discovered a second PD morphotype (type I) that lacks a visible cytoplasmic sleeve but is capable of non-targeted movement of macromolecules, which indicates that the size of PD pores undergoes dynamic changes. Therefore, the space of cytoplasmic sleeve might increase substantially under certain condition that allows the fitting of actin filaments.

Interestingly, it was shown that several other class I formins are also able to target to PD. Specifically, the closest homolog of AtFH2, namely AtFH1, is also able to associate with PD (Diao et al., 2018; Oulehlova et al., 2019). AtFH1 functions redundantly with AtFH2 in regulating the permeability of PD (Diao et al., 2018). Strikingly, it was shown that several rice class I formins are also able to target to PD (Diao et al., 2018), suggesting that targeting of class I formins to PD is an evolutionarily conserved strategy in plants. An interesting but yet-to-be-answered question is how the TMD of the PD-localized class I formins have evolved to enable their targeting to PD. This function may be linked to the fact that the membrane of PD has a unique phospholipid composition (Grison et al., 2015). Certainly, it could be possible that the TMD of those class I formins might have additional functions besides the membrane anchoring. In support of this speculation, a very recent report showed that TMD of *Arabidopsis thaliana* Plasmodesmata-located protein (PDLP) 5 is involved in the self-interaction of PDLP5 that is essential for PDLP5 to regulate cell-to-cell movement besides its role in membrane targeting (Wang et al., 2020).

**Figure 1:**
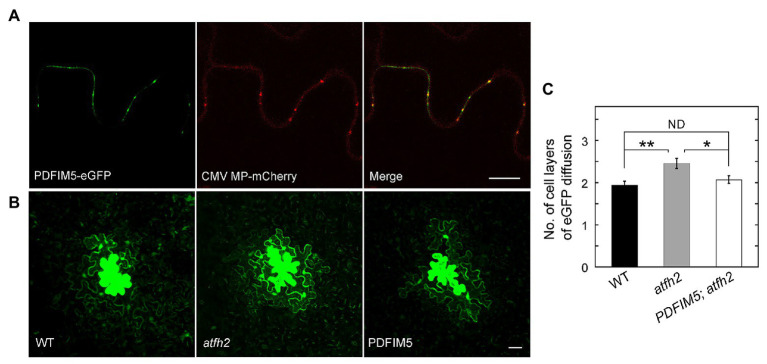
Targeting of FIMBRIN 5 (FIM5) to PD Alleviates the PD Phenotype in *atfh2* Mutants. **(A)** Subcellular localization of PDFIM5-eGFP and *Cucumber mosaic virus* (CMV) movement protein (MP)-mCherry in epidermal pavement cells of *Nicotiana benthamiana* leaves. PDFIM5 was obtained by fusing the N-terminal fragment of AtFH2 (AtFH2^N282^) with *Arabidopsis* FIMBRIN5 (Wu et al., 2010). PDFIM5 was further fused to eGFP (Diao et al., 2018). Plasmids encoding PDFIM5-eGFP and CMV MP-mCherry were introduced into *Agrobacterium tumefaciens* strain GV3101 and transiently expressed in *N. benthamiana* leaves by GV3101 injection. Bar = 10 μm. **(B)** Images of eGFP diffusion in leaf epidermal pavement cells of WT, *atfh2*, and *PDFIM5*; *atfh2* plants. PDFIM5 was constructed as in **(A)**. The PDFIM5 plasmid was introduced into *Agrobacterium tumefaciens* strain GV3101 and transformed into *atfh2* plants by the floral dip method. The PD permeability of WT, *atfh2*, and *atfh2* harboring PDFIM5 was assessed by the eGFP diffusion assay (Diao et al., 2019), and the images were collected by confocal microscopy. Bar = 10 μm. **(C)** Quantification of the number of cell layers with eGFP diffusion in *Arabidopsis* leaf epidermal pavement cells at 24 h after bombardment. *PDFIM5*; *atfh2* represents *atfh2* plants expressing PDFIM5. More than 30 cells were counted and the experiments were repeated at least three times. Error bars represent SE. ^*^p < 0.05 and ^**^p < 0.01 by Mann-Whitney U test. ND, no statistical difference.

As mentioned above, PD permeability is increased in *atfh2* mutants. Interestingly, targeting of *Arabidopsis* FIMBRIN 5 (FIM5) to PD alleviates the intercellular trafficking phenotype in *atfh2* mutants (Figure 1). This suggests that loss of AtFH2 causes instability of actin filaments and/or reduction in the amount of actin filaments in PD. These data actually support the previous notion that stabilization of actin filaments decreases the permeability of PD whereas destabilization of actin filaments increases it (Ding et al., 1996; Su et al., 2010). In summary, these data together suggest that the amount of actin filaments and/or the stability of actin filaments are crucial for the permeability of PD, and actin filaments in PD presumably act as the physical barrier to regulate the permeability of PD.

## Conclusions and Perspectives

Increasing evidence is showing that the actin cytoskeleton is involved in the regulation of intercellular transport through PD, whereas the molecular mechanism by which the actin cytoskeleton regulates the permeability of PD remains largely unexplored. Research in this area progresses slowly for at least two reasons. Firstly, researchers lack approaches to directly visualize the actin cytoskeleton in PD, because PD are tiny structures that are deeply embedded in the cell walls. Secondly, researchers lack approaches to specifically manipulate the function of the actin cytoskeleton in PD. Recent identification of PD-localized class I formins provides the possibility to manipulate the actin cytoskeleton in PD *via* regulating the function of those formins. Indeed, analysis of PD permeability in mutants lacking AtFH2 or AtFH1 and AtFH2, in combination with *in vitro* biochemical characterization of AtFH2, allows us to conclude that actin filaments might act as the physical barrier in controlling the permeability of PD. This is actually consistent with a previous assumption that actin filaments in PD might act as the filter in controlling PD permeability (Chen et al., 2010). However, the precise localization of AtFH2 in PD is currently unknown. Dissection of the AtFH2-mediated actin regulatory machinery in PD, for example, by searching for AtFH2-interacting proteins or screening for suppressors or enhancers of the *atfh2* mutant phenotype, might provide further insights into the function and regulation of the actin cytoskeleton in PD. In summary, recent characterizations of PD-localized class I formins have provided insights into the function and mechanism of action of actin in regulating the permeability of PD. However, it remains largely unknown how exactly the actin cytoskeleton regulates the structure and function of PD. This will be an exciting research avenue in the future.

## Author Contributions

All authors listed have made a substantial, direct and intellectual contribution to the work, and approved it for publication.

### Conflict of Interest

The authors declare that the research was conducted in the absence of any commercial or financial relationships that could be construed as a potential conflict of interest.
